# Breakpoint mapping and haplotype analysis of translocation t(1;12)(q43;q21.1) in two apparently independent families with vascular phenotypes

**DOI:** 10.1002/mgg3.346

**Published:** 2017-11-23

**Authors:** Tiia Maria Luukkonen, Mana M. Mehrjouy, Minna Pöyhönen, Anna‐Kaisa Anttonen, Päivi Lahermo, Pekka Ellonen, Lars Paulin, Niels Tommerup, Aarno Palotie, Teppo Varilo

**Affiliations:** ^1^ Institute for molecular medicine Finland FIMM University of Helsinki Helsinki Finland; ^2^ Department of Health National Institute for Health and Welfare Helsinki Finland; ^3^ Wilhelm Johannsen Centre for Functional Genome Research Department of Cellular and Molecular Medicine University of Copenhagen Copenhagen Denmark; ^4^ Clinical Genetics Helsinki University Hospital University of Helsinki Helsinki Finland; ^5^ Department of Medical Genetics University of Helsinki Helsinki Finland; ^6^ Laboratory of Genetics HUSLAB Helsinki Finland; ^7^ Institute of Biotechnology University of Helsinki Helsinki Finland; ^8^ Broad Institute of Harvard and MIT Cambridge MA USA

**Keywords:** balanced translocation, conserved haplotypes, mate pair sequencing, position effect, vascular phenotypes

## Abstract

**Background:**

The risk of serious congenital anomaly for de novo balanced translocations is estimated to be at least 6%. We identified two apparently independent families with a balanced t(1;12)(q43;q21.1) as an outcome of a “Systematic Survey of Balanced Chromosomal Rearrangements in Finns.” In the first family, carriers (*n* = 6) manifest with learning problems in childhood, and later with unexplained neurological symptoms (chronic headache, balance problems, tremor, fatigue) and cerebral infarctions in their 50s. In the second family, two carriers suffer from tetralogy of Fallot, one from transient ischemic attack and one from migraine. The translocation cosegregates with these vascular phenotypes and neurological symptoms.

**Methods and Results:**

We narrowed down the breakpoint regions using mate pair sequencing. We observed conserved haplotypes around the breakpoints, pointing out that this translocation has arisen only once. The chromosome 1 breakpoint truncates a *CHRM3* processed transcript, and is flanked by the 5′ end of *CHRM3* and the 3′ end of *RYR2*. *TRHDE*,*KCNC2*, and *ATXN7L3B* flank the chromosome 12 breakpoint.

**Conclusions:**

This study demonstrates a balanced t(1;12)(q43;q21.1) with conserved haplotypes on the derived chromosomes. The translocation seems to result in vascular phenotype, with or without neurological symptoms, in at least two families. We suggest that the translocation influences the positional expression of *CHRM3*,*RYR2*,*TRHDE*,*KCNC2*, and/or *ATXN7L3B*.

## 1 INTRODUCTION

Reciprocal balanced translocations are prevalent chromosomal aberrations with the incidence of *c*. 1:600 births. At least 6% of balanced translocations cause diseases (Warburton, 1991). Reciprocal translocations are nonrandomly distributed in the human genome due to selective advantage and more frequent potential of some DNA sequences to mediate breakage and recombination. The involvement of multiple mechanisms in the DNA breakage and repair process have been proposed. These mechanisms include (1) nonallelic homologous recombination (NAHR), (2) nonhomologous end joining (NHEJ), (3) microhomology‐mediated break‐induced replication (MMBIR), and (4) fork stalling and template switching (FoSTeS) (Gajecka et al., 2008; Gu, Zhang, & Lupski, 2008). Breakpoint analysis of disease‐associated rearrangements can elucidate the etiology and molecular mechanisms associated with disease phenotypes.

Cerebral infarction, or stroke, is a major cause of morbidity and death with a considerable economic burden to the modern society. It is a late‐onset, complex multifactorial disease; however, it can be a manifestation of a number of monogenic disorders that may boost the research toward treatments. Epidemiological studies and animal models strongly suggest genetic influences in the pathogenesis. Recent association studies on stroke have implicated several loci including *HDAC9* (OMIM *606543), *PITX2* (OMIM *601542), *ZFHX3* (OMIM *104155), *NINJ2* (OMIM *607297), 9q21, *KRTDAP* (OMIM *617212), *PLEKHA1* (OMIM *607772), *IL15* (OMIM *600554), *NDFIP2* (OMIM *610041), *SLC6A11* (OMIM *607952), *SLC6A9* (OMIM *601019), *NAA25* (OMIM *612755), and *ALDH2* (OMIM +100650) (Kilarski et al., 2014; Traylor et al., 2012).

Tetralogy of Fallot (TOF; OMIM 187500) is a rare (1/2,000), complex congenital heart defect manifesting with four classical structural defects: a ventricular septal defect, pulmonary stenosis, right ventricular hypertrophy, and overriding aorta. Symptoms include episodes of cyanosis as some of the oxygen‐poor blood fails to reach the lungs. TOF is associated with chromosome 22q11 deletion syndrome and trisomy 21, and it can be caused by heterozygous variants in *JAG1* (OMIM +601920), *NKX2‐5* (OMIM *600584), and *GATA4* (OMIM *600576) (Benson et al., 1999; Eldadah et al., 2001; Tomita‐Mitchell, Maslen, Morris, Garg, & Goldmuntz, 2007). Variants in *ZFPM2* (OMIM *603693), *GDF1* (OMIM *602880), *TBX1* (OMIM *602054), and *GATA6* (OMIM *601656) have been identified in sporadic TOF cases (Karkera et al., 2007; Maitra, Koenig, Srivastava, & Garg, 2010; Pizzuti et al., 2003; Rauch et al., 2010).

Translocations form through DNA double‐strand breakage (DSBs) followed by ligation repair processes. DNA DSBs with open DNA ends can be aligned and joined based on regional sequence homology through NAHR. NAHR is mediated by large tracks of LCRs or shorter homologous repetitive regions (Mefford & Eichler, 2009; Rudd et al., 2009; Sharp et al., 2006). DNA DSBs can also rejoin in the absence of sequence homology through NHEJ. NHEJ is a distinctive repair mechanism for its imprecision in leaving microalterations, “DNA scars,” which are seen at the breakpoint junctions of nonrecurrent rearrangements (Lieber, 2008; Pfeiffer, Goedecke, & Obe, 2000; Sargent, Brenneman, & Wilson, 1997; Yu & Gabriel, 2004). Recurrent translocations with clustered breakpoints arise through NAHR mediated by interchromosomal paralogous LCRs (Ou et al., 2011). NAHR between interspersed LINE and SINE/Alu elements can also result in translocations, which is further supported by Alu elements present in the LCR junctions (Bailey, Liu, & Eichler, 2003; Deininger, Moran, Batzer, & Kazazian, 2003; Elliott, Richardson, & Jasin, 2005). Identical Alu elements seem to mediate translocation formation through single‐strand annealing (SSA) repair, while diverged Alu elements seem to promote NHEJ repair (Elliott et al., 2005).

The majority of translocations are assumed to be unique (nonrecurrent), lacking significant breakpoint spanning sequence homology. To date, only a few recurrent translocations have been described in humans. The shared rearrangement‐prone breakpoint intervals of recurrent translocations provide cues to rearrangement formation. Recurrent t(11;22)(Kurahashi et al., 2000) and t(8;22)(Sheridan et al., 2010) are mediated by palindromic AT‐rich repeats. In t(8;22), the chromosome 8 palindromic AT‐rich repeat is flanked by two highly homologous Alu repeats that are in an inverted orientation with respect to one another. These Alu elements may also contribute to the translocation formation. Recurrent t(4;8) (Giglio et al., 2002), t(4;10)(van der Maarel et al., 2000), and t(4;11)( Ou et al., 2011; South et al., 2008) are mediated by low‐copy repeats (LCRs). The origin of potentially recurrent translocations can be determined by breakpoint mapping combined with haplotyping. Haplotype analysis enables differentiation between identical‐by‐descent (IBD) DNA segments at breakpoints versus genuinely recurrent breakpoints that have arisen more than once.

Balanced translocations affect the nuclear organization of chromosomes. The extent to which the spatial organization of the genome contributes to translocation formation has been investigated, but is not yet fully understood (Zhang et al., 2012). High‐resolution chromosome conformation capture (Hi‐C) can reveal topologically associating domains (TADs), which are chromatin compartments with physical interactions at conserved positions (Dixon et al., 2012). Disruption of TAD boundaries by translocations may be deleterious due to long‐range position effect caused by displacement of regulatory elements.

Chromosomal breakpoints have been used as signposts for critical genes in human disease (Johnson et al., 2006; Ray et al., 1985; Viskochil et al., 1990). The underlying pathogenetic mechanisms may be versatile, such as dosage‐sensitive break (single functional copy is insufficient for normal gene function), gene fusion (chimeric gene), position effect (dysregulation of gene expression through dislocation and disruption of noncoding elements such as RNA genes or conserved nongenic sequences; Dermitzakis, Reymond, & Antonarakis, 2005; Mattick, 2005), or unmasking of recessive variants (normal homologous chromosome becomes unmasked). The consequence may be haploinsufficiency, dominant‐negative or gain‐of‐function effect of a fusion protein, elevated expression, or complete absence of protein product when the other allele is imprinted or otherwise mutated.

In addition to an intact coding sequence, the spatially, temporally, and quantitatively correct gene expression requires regulatory control. In a variety of disease‐related cases, it is not the transcription unit but the altered regulation of gene expression that is affected. Such cases are categorized as “position effect.” When translocation separates the promoter or transcription unit from a regulatory element, it may reduce or silence the transcription, whereas removal of a silencer may lead to inappropriate activation of the gene. A translocation may also juxtapose a gene next to another regulatory element, or an euchromatic gene next to a region of heterochromatin, again resulting in inappropriate expression (Festenstein et al., 1996; Milot et al., 1996).

In this study, we utilized mate pair sequencing (MPS) and haplotyping to characterize the exact chromosomal breakpoints of a balanced t(1;12)(q43;q21.1) in two apparently independent families. The families suffer from vascular phenotypes including cerebral infarction and TOF.

## 2 MATERIALS AND METHODS

Informed consents approved by the Ministry of Social Affairs and Health and Ethical Committees of the Joint Authority for the Hospital District of Helsinki and Uusimaa were obtained for further characterization of the families and their chromosomal rearrangements.

### 2.1 Systematic Survey of Balanced Chromosomal Rearrangements in Finns

We have launched a national survey of balanced chromosomal rearrangements (BCRs) in Finns by ascertaining all known carriers (*n* = 3,016) from medical genetics departments and clinical genetics laboratories in Finland. Our database contains medical records of all available carriers including family members carrying the same rearrangement IBD. From this resource, we have drawn families with apparent correlating phenotype to the balanced translocation. In our gene‐mapping pilot, we identified a potential positional candidate gene for intracranial and aortic aneurysm. The survey and database are described more broadly in our prior publication (Luukkonen et al., 2012).

### 2.2 The International Breakpoint Mapping Consortium

In Denmark, the Wilhelm Johannsen Centre for Functional Genome Research, coordinated by Professor Niels Tommerup, has organized the International Breakpoint Mapping Consortium (IBMC) for the systematic mate pair mapping of ~10,000 constitutional BCRs. Currently, 160 research institutions have joined the consortium. The focus of the consortium project is on both post‐ and prenatally diagnosed BCRs, on de novo and familial BCRs, and on BCRs associated with both affected and unaffected individuals. The aim is to enroll ~4,500 subjects who have previously been diagnosed with constitutional apparently balanced chromosomal translocations or inversions.

MPS, utilized by the IBMC project, has dramatically simplified high‐throughput mapping of BCRs. As BCRs are heterogeneous, an optimal exploitation requires both the concerted effort and large number of participating institutions. When achieved, the huge number of accessible BCRs will become a unique resource for the identification of human genotype–phenotype associations.

### 2.3 Cytogenetic analysis

Standard cytogenetic analyses were performed to verify earlier karyotyping. Blood samples were obtained from individuals A1, A2, and B1. G‐banding karyotype analysis was performed from peripheral blood lymphocytes according to standard protocols.

### 2.4 Genome‐wide mate pair sequencing

To identify the exact translocation breakpoints, we used next‐generation MPS method on genomic DNA extracted from the blood lymphocytes of patients A1, A2, and B1. For individuals A1 and A2, mate pair libraries were prepared using 5500 SOLiD™ Mate‐Paired Library Kit (Thermo Scientific, Waltham, MA, USA) at DNA Sequencing and Genomics Laboratory, Institute of Biotechnology, University of Helsinki. The DNA integrity was evaluated by agarose gel electrophoresis, and concentration was quantified using Invitrogen Qubit™ fluorometer (Thermo Scientific). Genomic DNA (5 μg) was sheared using the Covaris™ System (Covaris, Inc., Woburn, MA, USA) to yield average 1.5 kb fragments. After circularization, nick‐translation, end‐repair, and A‐tailing Illumina TruSeq adapters were ligated. Final mate pair libraries were size selected to ~500 bp fragments and were subjected to 2 × 150 base paired end sequencing using Illumina HiSeq2500 Rapid Run sequencing platform.

For individual B1, mate pair library was prepared at the Wilhelm Johannsen Centre for Functional Genome Research, Denmark. The integrity was evaluated by agarose gel electrophoresis and concentrations measured by Nanodrop ND‐1000 spectrometer (Thermo Scientific). Nextera mate pair libraries were constructed using 1 μg of DNA following the instruction for a gel‐free preparation of 2 kb effective insert size Mate Pair Library v2 (Nextera Mate Pair Library Prep; Illumina, San Diego, CA, USA). Final libraries were quantified using Pico Green (Quant‐iT; Invitrogen). Four different indexed libraries were pooled together with other samples and sequenced on a single lane of the HiSeq2500 (Illumina) (2 × 100 bp).

### 2.5 Data processing

#### 2.5.1 Patients A1 and A2

Raw sequence reads were quality checked using FastQC. Reads were processed using Cutadapt against Illumina TrueSeq adaptors and circularization adaptor. Sequence bases with phred score quality <10 were trimmed, and reads shorter than 20 bp were discarded. Processed read sets were quality checked by FastQC, and then aligned to the human reference genome (GRCh37/hg19/GCA_000001405.1) using NovoAlign V2 software (Novocraft Technologies Sdn Bhd, Selangor, Malaysia) with quality calibration and Mate Pair mode. Picard mark duplicates was performed on the merged BAM file for each set. Alignments generated by NovoAlign were analyzed with Samtools genotyper (http://samtools.sourceforge.net) to call for single‐nucleotide variants and microshort indels. The variants called were then annotated with SnpEff (http://snpeff.sourceforge.net) and Ensembl Variant Effect Predictor (VEP) (http://www.ensembl.org/info/docs/tools/vep). For structural variant (SV) detection, BAM files were run through Breakdancer (http://breakdancer.sourceforge.net) and Pindel (Ye, Schulz, Long, Apweiler, & Ning, 2009), and SVs were annotated using VEP. From the processed uniquely mapping read pairs, we searched for those spanning the breakpoints. Read pairs aligning with unexpected orientation or distances were defined as discordant. We used the integrative genomics viewer (IGV) (Broad Institute, Cambridge, Massachusetts, USA) to visualize discordant reads aligning to different chromosomes and find the approximate region of the breakpoints.

#### 2.5.2 Patient B1

Raw sequence reads were first trimmed and then Cutadapt was used to remove the adaptors. The remaining pairs passing Illumina Chastity filtering (>0.6) were mapped to the human reference genome (GRCh37/hg19) using Burrows Wheeler Aligner (BWA) (Li & Durbin, 2010). To annotate the unique SVs, reads not aligning uniquely, were removed and the ones with unexpected orientation or aligning to different chromosomes were extracted using SVDetect (http://svdetect.sourceforge.net/)(Zeitouni et al., 2010) and Delly (www.korbel.embl.de/software.html). The predicted SVs were compared with multiple in‐house mate pair datasets to identify sample‐specific SVs. By uploading the annotated BED files into IGV (Broad Institute), we visualized the SVs and found the approximate region of the breakpoints. The depth of coverage of the aligned mate pair reads was used to detect copy number changes.

In all patients, confirmatory screening across the translocation junction fragments, suggested by MPS, was conducted using Sanger sequencing. To identify interspersed repetitive elements near the breakpoints, we referred to the UCSC Genome Browser (GRCh37/hg19).

### 2.6 Exclusion of relatedness

To exclude any unknown relatedness, individuals A1 and B1, and additional 700 controls representative of the general Finnish population, were genotyped at the Institute for Molecular Medicine Finland (FIMM) Technology Centre using the Illumina Human CoreExome‐24_v1‐1_A (Illumina). Relatedness was determined using the IBD calculation and multidimensional scaling (MDS) on PLINK whole genome association analysis toolset version 1.07 (Purcell, 2007).

### 2.7 Haplotype analysis

We applied haplotype analysis to determine the origin of t(1;12)(q43;q21.1) in our families. We genotyped one individual per family using the Illumina Human CoreExome‐24_v1‐1_A (Illumina) at the FIMM Technology Centre. For the IBD shared segment detection, we used the Beagle software packages v4.0 and v4.1 (Browning & Browning, 2013). We pruned the genotype data using PLINK v1.9 software to exclude SNPs with pairwise genotypic *r* ^2^ > 50% within sliding windows of 100 SNPs with a 25‐SNP increment between windows (Purcell, 2007). For the replication haplotype analysis, we used FISHR2 IBD segment detection program (Bjelland, Lingala, Patel, Jones, & Keller, 2017). The used settings for this software allowed no gaps in the shared segments. The suitability of the used –emp‐pie‐threshold value 0.015 and –emp‐ma‐threshold value 0.045 were tested with the parameter_finder tool included in the FISHR2 package and were found to be very conservative and ruling out the majority of false positives in this material. Prior to FISHR2 analysis, the SNP data were phased using SHAPEIT (Delaneau, Marchini, & Zagury, 2011).

## 3 RESULTS

### 3.1 Cytogenetic analysis

**Figure 1:**
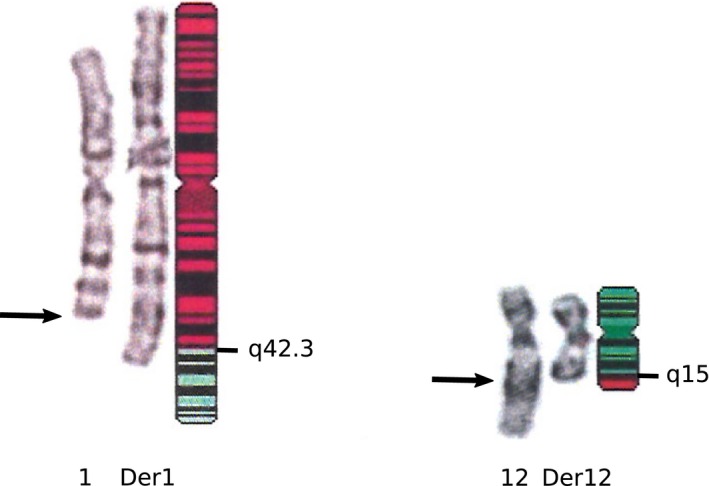
Ideogram with the breakpoint regions indicated

Standard karyotype analysis displayed a balanced translocation t(1;12)(q42.3;q15) for patients A1 and A2, and t(1;12)(q42;q15) for patient B1 (Figure 1).

### 3.2 Clinical summary

#### 3.2.1 Family A

The proband contacted us with concern that the balanced translocation t(1;12) causes medical problems in their family. The proband, all three children, and two of five grandchildren carry the translocation and have similar symptoms, which no‐one else manifests in their family. As a child, they had specific learning problems. As a middle aged, they suffer from a chronic headache, balance problems, tremor, and fatigue. The oldest retired early, and the two oldest, the proband and the eldest child, have had strokes at the ages of 53–58. The youngest child has been hospitalized suspected of TIA at the age of 44. The oldest grandchild has been examined because of hyperkinetic disturbance of attention, and the youngest for special difficulties in development and attention. Both grandchildren have been rehabilitated and are progressing well to normal school. In their extensive examinations, the neurologists, the otorhinolaryngologists, the neuropediatricians, the psychologists, the general practitioners, and the clinical geneticists have often referred to the translocation as causal but failed to set a specific diagnosis. There is no predisposing alcohol, tobacco, cholesterol, coagulation factor, nor neurological, psychiatric, or otorhinolaryngological disease.

#### 3.2.2 Family B

**Figure 2:**
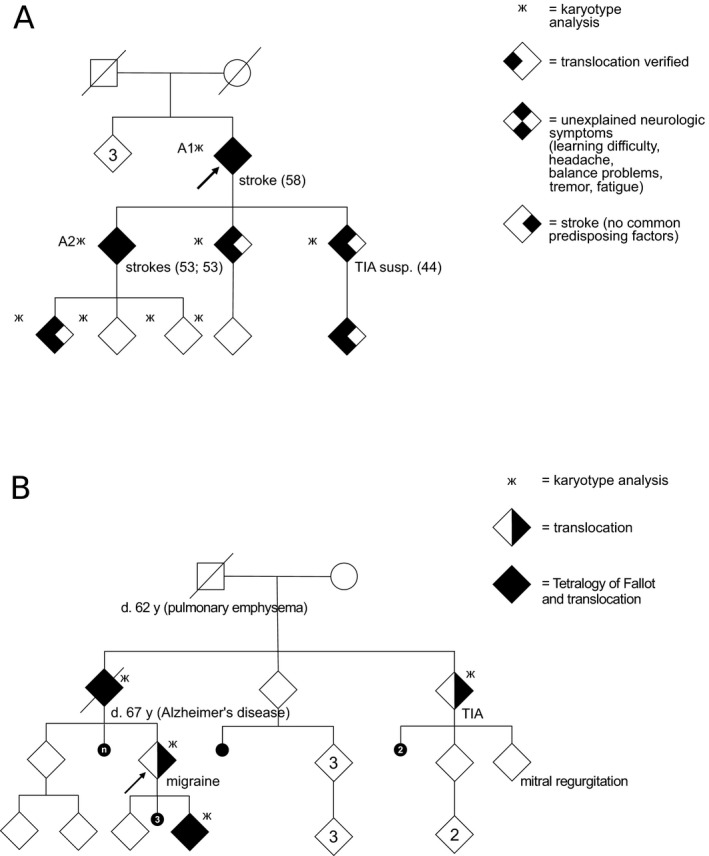
The pedigrees of the families. In the pedigree (a), carriers (*n* = 6) manifest first with learning problems in childhood and later with neurological symptoms (chronic headache, balance problems, tremor, fatigue), TIA, and cerebral infarctions in their fifties without identified predisposing factor. The numbers A1 and A2 in the pedigree refer to the patient numbers in the text, patient A1 had a stroke at the age of 58, and patient A2 had two strokes at the age of 53. In the pedigree (b), two carriers suffer from tetralogy of Fallot, one from TIA and one from migraine

The proband sought for doctor's appointment because of recurrent miscarriages. The proband had migraine, but was otherwise healthy. The karyotype analyses revealed a balanced translocation t(1;12) segregating in the family. Soon the proband conceived for their second child. The fetus had inherited the translocation and had TOF. The proband's parent had also TOF and carried the translocation. The grandparents are not known to have had heart problems, but the great grandparent died under 40 years of age and delivered a dysmorphic child, who deceased neonatally. The proband's parent's sibling is a carrier of the translocation and has suffered from TIA, hypertension, and two miscarriages with the spouse. Their second child has mitral regurgitation and arrhythmia, but no karyotype analysis has been performed and is not available for analysis. The pedigrees of the families are given in Figure 2.

### 3.3 Breakpoint mapping

**Figure 3:**
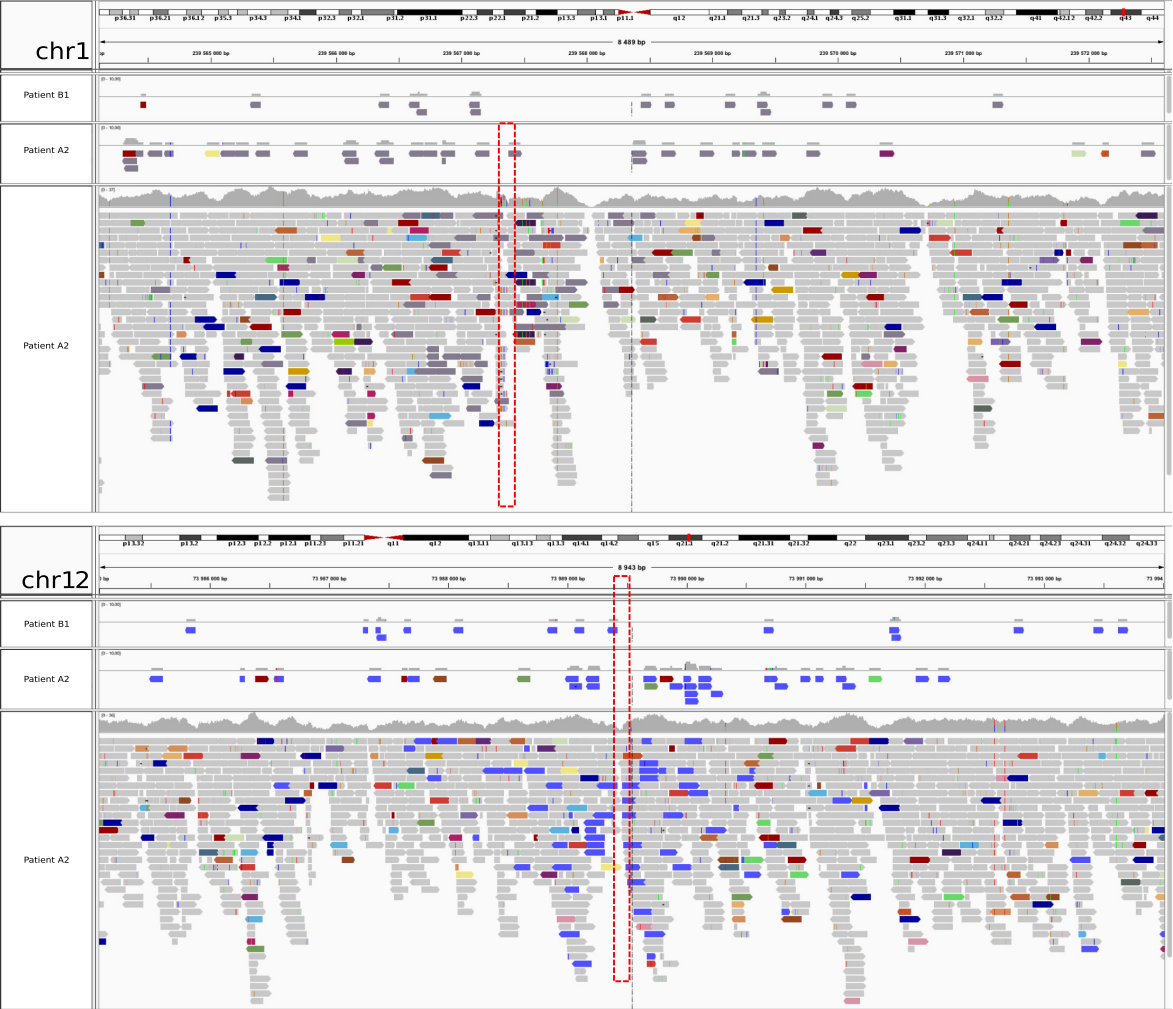
The discordant mate pair reads between chromosomes 1 and 12. (a) Dark gray indicates reads with mate end sequence on chromosome 12 and (b) blue indicates reads with mate end sequence on chromosome 1. In (a) and (b), the two upper most panels display solely the discordant reads for patients B1 and A2, whereas the bottom panel displays all aligned reads for patient A2

**Figure 4:**
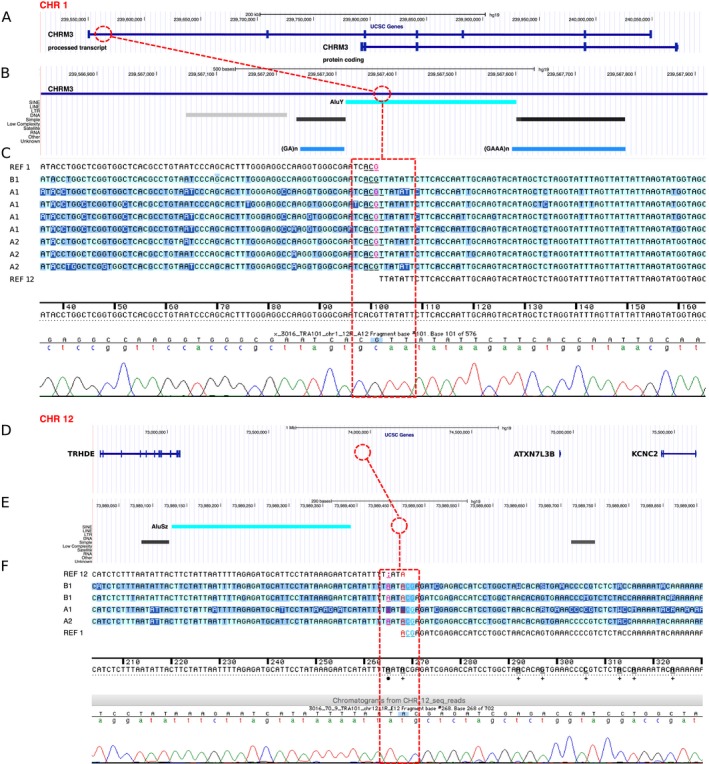
The chromosome breakpoints (connecting dashed line) adapted from the UCSC Genome Browser (http://genome.ucsc.edu) (a, b, d, e) and the Sequencher 5.4.5 Software (c, f). An AluY element (=bright turquoise) is intersecting the chromosome 1 breakpoint (b), and an AluSZ (=bright turquoise) element lies 71 bp downstream of the chromosome 12 breakpoint (e). The chromosome 1 breakpoint is flanked by the 3′ end of *CHRM3* (NG_032046.2, 225 kb). On the chromosome 12, *TRHDE* (NG_046971.1, 930 kb), *KCNC2* (NC_000012.12, 1.5 Mb), and *ATXN7L3B* (NC_000012.12, 942 kb) are adjacent to the breakpoint. The simple repeats, (GA)n (size = 83 bp) and (GAAA)n (size = 174 bp) on the chromosome 1 are indicated in light blue (b)

**Figure 5:**
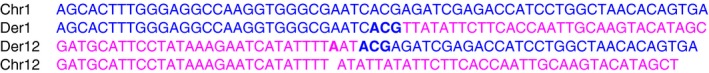
The translocation breakpoint junction fragment sequences. The reference sequences are labeled in blue and pink for the chromosome 1 and the chromosome 12, respectively. A single base pair insertion “A” is bolded in pink in the der(12). An “ACG” duplication is bolded in blue in the der(1) and the der(12). Chr, chromosome; Der, derivative chromosome

We refined the translocation breakpoints based on the MPS findings to t(1;12)(q43;q21.1) for patients A1 and A2, and to t(1;12)(q43;q21.1) for patient B1. We narrowed down the breakpoint regions to 6 bp on chromosome 1, and 42 bp on chromosome 12, defined by the overlapping read pairs (Figure 3a,b). Sanger sequencing mapped the exact breakpoint positions to chr1:239,567,377–239,567,379 and chr12:73,989,462–73,989,463 (Figure 4). The translocation breakpoint positions and the surrounding genomic architecture were identical at base pair level in all patients (Figure 5). Analysis of the flanking sequences revealed a breakpoint intersecting SINE/AluY element on chromosome 1, and a SINE/AluSz element in the close vicinity of the chromosome 12 breakpoint (71 bp upstream) (Figure 4b,e). Two simple repeats, (GA)n (size = 83 bp) and (GAAA)n (size = 174 bp) map 63 bp upstream and 232 bp downstream from the chromosome 1 breakpoint, respectively (Figure 4b). We detected no apparent regional microhomology between the breakpoint junctions. We identified a breakpoint flanking microduplication and an insertion: the chromosome 1 breakpoint contained a three base pair duplication of “ACG” present in both derivative chromosomes, and the chromosome 12 breakpoint a single base pair insertion of “A” present on the derivative chromosome 12 (Figure 5). Both breakpoints are intergenic. The chromosome 1 breakpoint truncates a *CHRM3* processed transcript, and is flanked by two protein‐coding genes at 1q43: the 5′ end of a protein coding *CHRM3* (OMIM *118494) (225 kb upstream) and the 3′ end of *RYR2* (OMIM *180902) (1.6 Mb downstream). *TRHDE* (OMIM *606950) (930 kb upstream), *KCNC2* (OMIM *176256) (1.5 Mb downstream), and *ATXN7L3B* (OMIM *615579) (942 kb downstream) are located closest to the chromosome 12 breakpoint (Figure 4a,d).

### 3.4 Exclusion of relatedness

**Figure 6:**
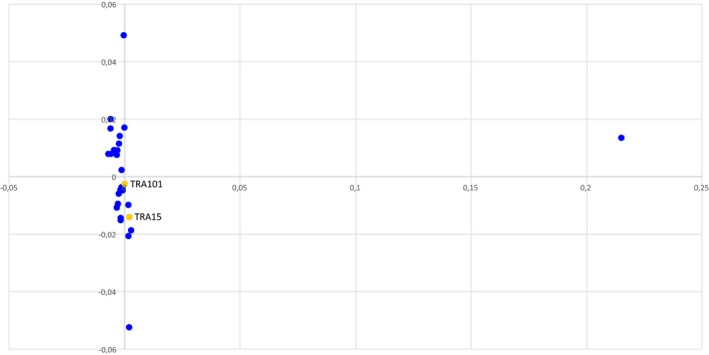
The multidimensional scaling analysis (MDS) scatter plot of the patients A2 and B1 (yellow dots) and 30 randomly selected controls (representative of the general Finnish population) (blue dots) excludes relatedness

We excluded relatedness between the two families carrying the identical balanced t(1;12)(q43;q21.1) by performing IBD testing and MDS. The estimated PI_HAT value for the patients A2 and B1 was 0.0006. See Figure 6 for the MDS plot.

### 3.5 Haplotype analysis

**Figure 7:**
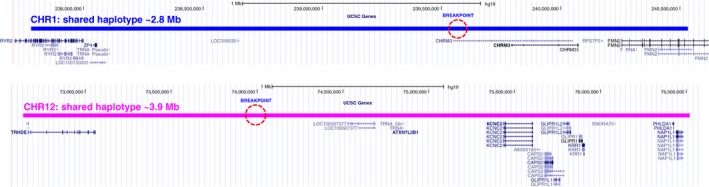
Shared segments of IBD for the translocated chromosomes. The breakpoints are indicated with dashed circles. The chromosome 1 segment is ~2.8 Mb in size, and the chromosome 12 segment is ~3.9 Mb in size

The t(1;12)(q43;q21.1) in the two apparently independent families was IBD with identical breakpoint intervals and conserved haplotypes. Based on the Beagle v4.1 analysis, the shared haplotype extends ~1.8 Mb upstream and 1 Mb downstream from the chromosome 1 breakpoint (LOD score = 6.59; LOD score threshold for IBD segment = 3.0). For chromosome 12, the haplotype extends 1.4 Mb upstream and 2.5 Mb downstream from the breakpoint (LOD score = 9.12). The breakpoint flanking shared segments of IBD are presented in Figure 7. Based on the FISHR2 replication analysis, the shared haplotype extends ~6.7 Mb upstream and 0.9 Mb downstream from the chromosome 1 breakpoint. And for chromosome 12, the haplotype extends ~15.6 Mb upstream and ~2.5 Mb downstream from the breakpoint.

## 4 DISCUSSION

The translocation breakpoints mapped outside the putative protein‐coding genes (i.e., transcription unit and promoter region remain intact), pointing to malfunction caused by a position effect. As the translocation t(1;12)(q43;q21.1) disrupts the *CHRM3* processed transcript at 1q43, we postulate that it may affect potassium channel mediation in the brain through compromised *CHRM3* function. Dysfunction may be induced via truncation of the *CHRM3* processed transcript with a potential role in the regulation of protein coding *CHRM3* expression, or alternatively via position effect triggered by disruption of a relevant regulatory element.

The chromosome 1 breakpoint at 1q43 is flanked by two genes: cholinenergic receptor muscarinic 3 (*CHRM3*, 225 kb upstream) and ryanodine receptor 2 (*RYR2*, 1.6 Mb downstream). Chromosome band 1q42 has previously emerged in a linkage study suggesting a susceptibility gene for episodic ataxia type III (EA3) (Cader, Steckley, Dyment, McLachlan, & Ebers, 2005). EA3 is a very rare form with vestibular ataxia, vertigo, tinnitus, and myokymia (Steckley, Ebers, Cader, & McLachlan, 2001) (Cader et al., 2005). *CHRM3* encodes a muscarinic acetylcholine receptor M3 distributed widely throughout the body, for example, in the smooth muscles, the endocrine and exocrine glands, the lungs, the pancreas, and the brain. This receptor belongs to a larger family of G protein‐coupled receptors whose functions include adenylate cyclase inhibition, phosphoinositide degeneration, and modulation of potassium channels through the action of G proteins. Muscarinic M3 receptors are expressed in the hypothalamus and the dorsal vagal complex of the brainstem, regions of the brain that regulate insulin homeostasis. Their activation causes smooth muscle contraction and secretion from glandular tissue. Transmembrane potassium transport contributes fundamentally to the regulation of the membrane potential and neuronal signaling and prevents neuronal hyperexcitability, which in turn is believed to trigger ataxia and seizures.

The ryanodine receptor 2 (RYR2) found in cardiac muscle sarcoplasmic reticulum functions as a component of a calcium channel that supplies calcium to cardiac muscle. RYR2 has an essential role in embryonic cardiogenesis. Its mutations have been associated with stress‐induced polymorphic ventricular tachycardia and arrhythmogenic right ventricular dysplasia. RYR2 is most abundantly expressed in the brain, while all three isoforms are highly expressed in the cerebellum, hippocampus, olfactory region, basal ganglia, and cerebral cortex. The cerebral expression of RyRs is under tight regulation undergoing changes throughout development from embryonic to adult. Altered calcium signaling and RyR dysregulation have been implicated in neurodegenerative disorders such as Parkinson's disease and Alzheimer's disease (Abu‐Omar, Das, Szeto, & Feng, 2017).

*TRHDE*,* KCNC2*, and *ATXN7L3B* are genes located closest to the chromosome 12 breakpoint. *TRHDE* encodes a thyrotropin‐releasing hormone (TRH) degrading enzyme that specifically cleaves and inactivates the neuropeptide TRH. TRH is thought to modulate and normalize the central nervous system activity through its neuromodulatory functions that affect neuronal excitability (Metcalf & Dettmar, 1981). TRH and some of its stable analogs improve neurologic dysfunctions such as neuronal death occurring after TIA (Shishido et al., 1999). TRH therapy is used in several neurologic disorders, including spinocerebellar ataxia (SCA) where the improvement of ataxic gait is one of the important pharmacological responses (Sobue et al., 1983; Urayama, Yamada, Kimura, Zhang, & Watanabe, 2002). *TRHDE* is mainly expressed in the brain, including cerebellar hemisphere, frontal cortex, cerebellum, and cortex (http://www.gtexportal.org/home/gene/TRHDE). Herein, we propose that the chromosome 12 translocation breakpoint flanked by the 3′ end of *TRHDE* may cause inappropriate activation of *TRHDE* through a position effect, subsequently, potentially leading to excess inactivation of TRH in the cerebellum.

*KCNC2* encodes a potassium voltage‐gated channel subfamily C member 2 (K_V_3.2), which belongs to the delayed rectifier class of channel proteins and is an integral membrane protein that mediates the voltage‐dependent potassium ion permeability of excitable membranes. *ATXN7L3B* is a single exon putative ataxin‐7‐like 3B gene of unknown function. In their recent report, Rajakulendran, Roberts, Koltzenburg, Hanna, & Stewart (2013) described the clinical and genetic findings in a family with a 670 kb deletion of chromosome 12q21 and neurodevelopmental delay with cerebellar ataxia. *KCNC2* and *ATXN7L3B*, lying within the deleted region, were suggested to have a potential role in the complex neurodevelopmental and ataxic phenotype of the family. Variants in *KCNC3* (K_V_3.3), a member of the same subfamily closely related to *KCNC2*, have previously been associated with SCA13 (Herman‐Bert et al., 2000; Parolin Schnekenberg et al., 2015; Pyle et al., 2015; Waters et al., 2005, 2006). Variants in another potassium channel gene, *KCNA1* (K_V_1.1), underlie EA1 (Browne et al., 1994). Ataxin gene variants in *ATXN1*,* ATXN2*,* ATXN3*, and *ATXN7* cause SCA1, SCA2, SCA3, and SCA7, respectively. *ATXN7L3B* is highly expressed in the brain, particularly cerebellar hemisphere, frontal cortex, cerebellum, and hypothalamus (http://gtexportal.org/home/gene/ATXN7L3B). However, there are no data on the *ATXN73B* function, thus the potential pathogenic role would require identification of variants in further families with similar phenotypes. *KCNC2* is also mainly expressed in the brain, particularly frontal cortex, anterior cingulate cortex, cortex, hypothalamus, pituitary, and hippocampus (http://www.gtexportal.org/home/gene/KCNC2).

We examined the genomic architecture of both breakpoint regions. Analysis revealed two types of junction spanning sequence changes likely originating from the rearrangement formation, including a single base pair insertion “A” in der(12), and a trinucleotide “ACG” duplication present in both der(1) and der(12). We detected no apparent regional microhomology between the breakpoint junctions. However, carriers exhibit a breakpoint spanning AlyY element in the chromosome 1, and an AluSz element mapping 71 bp upstream from the chromosome 12 breakpoint. The identified Alu elements, with a 68.7% sequence similarity, may have mediated formation of the translocation via illegitimate homologous unequal recombination. The lack of higher level regional microhomology, proximity of the diverged Alu elements, and insertion of the “DNA scars” at sites of repair, implies NHEJ as an optional mechanism of translocation formation (Jeggo, 1998; Pfeiffer et al., 2000; Sargent et al., 1997).

Here, we have described two families, unrelated by IBS calculation and MDS analysis, carrying a t(1;12)(q43;q21.1). Interestingly, conserved haplotypes around the breakpoints suggest that in even apparently independent families similar translocations may arise only once (with the exception of reported recurrent translocations). The putative genes, lying some distance from the breakpoints, have previously been associated with similar clinical manifestations. We suggest that the translocation influences the positional expression of *CHRM3*,* RYR2*,* TRHDE*,* KCNC2*, and/or *ATXN7L3B*. The underlying mechanism may be the disruption of a regulatory element, resulting in induced or reduced gene expression and causing the vascular phenotypes and neurological manifestations. However, the translocation may cause a position effect even further upstream or downstream from the breakpoint.

Our goal is to scrutinize the genetic basis of clinical phenotypes in carriers of apparently balanced translocations. Chromosomal rearrangements are valuable signposts for genes and regulatory loci in human disease, thus they serve as an important resource for discovery and annotation. Analysis of MPS data allows precise breakpoint detection and further refinement of previously identified balanced and unbalanced rearrangements, moreover, pinpointing a causal gene for some patients. Due to its longer insert sizes, MPS facilitates mapping across repetitive regions and large introns.

In conclusion, we identified two apparently independent families manifesting with vascular phenotypes and neurological symptoms, and a translocation, in which breakpoint formation is likely to be Alu mediated. The translocation appears to produce the vascular phenotypes with a certain degree of similarity, yet unique features are present as well. The complex neurological symptoms, including learning problems, chronic headache, balance problems, tremor, and fatigue may be manifestations of the underlying vascular disease affecting the brain. The observed divergence in symptoms may be explained by variation in phenotypic penetrance and expressivity due to different combinations of modifying alleles and/or environmental factors between the families. Further characterization of the vascular phenotype and neurological symptoms, including stroke and TOF, requires identification of additional families with disruption of the chromosomal regions reported in this study.

## CONFLICT OF INTEREST

None declared.
